# Likability’s Effect on Interpersonal Motor Coordination: Exploring Natural Gaze Direction

**DOI:** 10.3389/fpsyg.2017.01864

**Published:** 2017-10-26

**Authors:** Zhong Zhao, Robin N. Salesse, Ludovic Marin, Mathieu Gueugnon, Benoît G. Bardy

**Affiliations:** ^1^Institute of Human Factors and Ergonomics, Shenzhen University, Shenzhen, China; ^2^EuroMov, University of Montpellier, Montpellier, France; ^3^Institut Universitaire de France, Paris, France

**Keywords:** motor coordination, likability, gaze direction, interpersonal relationship, eye tracking

## Abstract

Although existing studies indicate a positive effect of interpersonal motor coordination (IMC) on likability, no consensus has been reached as for the effect of likability back onto IMC. The present study specifically investigated the causal effect of likability on IMC and explored, by tracking the natural gaze direction, the possible underlying mechanisms. Twenty-two participants were engaged in an interpersonal finger-tapping task with a confederate in three likability conditions (baseline, likable, and unlikable), while wearing an eye tracker. They had to perform finger tapping at their comfort tempo with the confederate who tapped at the same or 1.5 times of the participant’s preferred frequency. Results showed that when tapping at the same frequency, the effect of likability on IMC varied with time. Participants coordinated at a higher level in the baseline condition at the beginning of the coordination task, and a facilitative effect of likability on IMC was revealed in the last session. As a novelty, our results evidenced a positive correlation between IMC and the amount of gaze onto the coordination partner’s movement only in the likable condition. No effect of likability was found when the confederate was tapping at 1.5 times of the participant’s preferred frequency. Our research suggests that the psychosocial property of the coordinating partner should be taken into consideration when investigating the performance of IMC and that IMC is a parameter that is sensitive to multiple factors.

## Introduction

In social interaction, psychological processes and behavioral activities are highly involved simultaneously. People verbally communicate with each other, appreciate the likability of the interaction partner, and behaviorally coordinate with the person. The present study aimed at exploring whether the likability of an individual would influence interpersonal motor coordination (IMC).

Likability refers to the degree of preference of a target individual by another individual (Reysen, 2005), and it indicates the quality of the interpersonal relationship. Literature also refers to affiliation and rapport as synonyms of likability (Bernieri, 1988; Hove and Risen, 2009; Miles et al., 2011).

In the current paper, we adopted Bernieri and Rosenthal’s (1991) definition of IMC, which can be broadly classified as behavioral matching and interactional synchrony(Bernieri and Rosenthal, 1991). Behavioral matching, also known as behavioral mimicry, refers to the phenomenon that individuals adopt the postures, gestures, and mannerisms of interaction partners (Chartrand and Bargh, 1999). Interactional synchrony mainly emphasizes the congruency in the temporal aspects of behavior, and illustrates how two people act simultaneously (Bernieri et al., 1988). Behavioral mimicry and interpersonal synchrony are regular forms of IMC. A large body of research from social psychology, neuroscience, and coordination dynamics indicates that during social interaction individuals do not act independently from each other; instead, their movements coordinate as long as there is a perceptual contact (Bernieri, 1988; Bernieri et al., 1988; Bernieri and Gillis, 1995; Schmidt and O’Brien, 1997; Schmidt et al., 2012).

Motivated by the notion that human behavior and psychological states are tightly intertwined with each other, the relation between IMC and likability has attracted a good amount of research interest. Already in the 1960s, psychologists were intrigued by the correspondence between level of mimicry and likability of partners in interaction. For instance, Charny (1966) reported a positive correlation between postural congruency and rapport between the psychotherapist and the client (Charny, 1966). Strong correlation was also found between IMC and teacher-student rapport (Bernieri, 1988). Recent research analyzed video clips of interactions between therapists and patients (Ramseyer and Tschacher, 2011) and found that non-verbal synchrony was associated with the outcome of the therapy, suggesting a positive correlation between IMC and likability.

Beyond a simple correlation between IMC and likability, past research has also suggested that IMC leads to higher level of likability between interactants. Chartrand and Bargh (1999) reported that the group of participants who were mimicked by the confederate liked the confederate more as compared to the group who was not mimicked (Chartrand and Bargh, 1999), suggesting that mimicry facilitates likability. Lakin and Chartrand (2003) also observed that people who attempted to affiliate with the partner mimicked the person more, inferring that mimicry might be an unconscious vehicle individuals utilize to achieve the purpose of being affiliated with others during social interactions (Lakin and Chartrand, 2003). Hove and Risen (2009) even demonstrated the existence of a causal effect of IMC onto likability. By adopting a finger-tapping task, they obtained a positive correlation between likability and IMC, but more importantly, found that likability was significantly higher in a synchronous condition compared to asynchronous and control conditions. Finally, they showed that likability was higher when synchronizing with another human than with an inanimate object, suggesting that likability arises from interpersonal relationship (Hove and Risen, 2009).

Existing research has therefore reached a common agreement on a positive relation between likability and IMC and as a consequence, it can be claimed that IMC would lead to likability increase. However, although previous research has shown a causal relationship from IMC to likability, no studies have yet proven a causal relationship from likability to IMC. Several previous studies suggest that this may be the case. For example, in order to seek whether social context would modulate how people coordinate with each other, Miles et al. (2010) manipulated the confederate’s punctuality or tardiness, and found a lower degree of IMC with the tardy confederate (Miles et al., 2010), indicating that manipulating likability induced IMC changes. The work conducted by Cesario et al. (2006) provided evidence that mimicry is modulated in some way by the likability of the interacting partner (Cesario et al., 2006). Recent studies also found that a divergence of arguments between interactants can disrupt in-phase bodily coordination (Paxton and Dale, 2013).

All of these studies support the idea that the level of bodily coordination is influenced by the likability of the interaction partner. However, because IMC can be used as a means to establish rapport (Chartrand and Bargh, 1999; Lakin and Chartrand, 2003; Hove and Risen, 2009), possibility remains that individuals would coordinate at a higher level with the unlikable individual when they desire to be affiliated with this person. This idea was supported by the study conducted by Miles et al. (2011), who explored whether group membership influenced IMC. They found a higher percentage of in-phase coordination with the out-group compared to the in-group confederate. This study inferred that individuals were more coordinated with members of the out-group in order to gain likability and search for affiliation, suggesting that low levels of current likability may lead to higher IMC if the interlocutors are trying to bond with one another (Miles et al., 2011). Similarly, Lakin et al. (2008) found that participants coordinated more with individuals who’ve just ostracized them, and this study also suggested the possibility of coordinating more with an unlikable person (Lakin et al., 2008).

Therefore as the main objective of the present study, we were particularly interested in seeking the causal effect of likability on IMC. We reasoned that if this was true, then even with the same interacting partner, higher level of IMC might be witnessed with higher likability, and lower IMC with lower likability. Moreover, we attempted to investigate the role of gaze in the relation between likability and IMC.

Our study was conceived in the theoretical framework of the dynamical approach to IMC. In this context, IMC is a self-organized phenomenon, which follows basic dynamic principles (Schmidt et al., 1990; Schmidt and O’Brien, 1997; Richardson et al., 2007). The majority of these studies required participants to perform rhythmic oscillatory movement. Each single individual was considered as an oscillator, and the level of IMC depended on the level of entrainment between the two oscillators. The above-mentioned relation between likability and IMC suggested that likability might influence the strength of the entrainment. But once again it still remains an open question whether likability would increase or decrease IMC. The present study specifically aimed to address the following two questions:

(1) Does increased likability causally enhances IMC, and does decreased likability causally decrease IMC?
(2) If likability leads to IMC change, are those changes mediated by directing more gaze toward the partner’s movement?

For (1) we emphasized that if a causal relationship exists, IMC would follow the change of likability. To fulfill that purpose, we arranged participants to interact with a confederate whom they had not known before the experiment started. Conversations were arranged to manipulate the likability toward the confederate. Interpersonal finger-tapping task was adopted right after the conversation. Participants had to tap with their index finger while the confederate was performing the same movement in their visual field. We expected that the coordination level would be higher in the likable condition.

For (2) gaze toward the partner’s movement was hypothesized as a mediator between likability and IMC, for several reasons. First, IMC cannot be possibly established without perception. Although coordination can be established via a variety of different perceptual modalities [e.g., visual (Schmidt and O’Brien, 1997; Richardson et al., 2007), auditory (Shockley et al., 2003; Baumann et al., 2007), tactile (Marin et al., 2009)], here we only focused on the role that visual perception played in establishing IMC. The perceptual basis of IMC has been confirmed by studies adopting both intentional (Schmidt et al., 1990) and unintentional motor coordination (Schmidt and O’Brien, 1997; Oullier et al., 2008). Second, the amount of available visual information is positively correlated to the level of entrainment in unintentional rhythmic coordination. For instance, Richardson et al. (2007) tested whether the extent to which participants fixated the partner’s movement influenced the level of coordination. During unintentional coordination, they found a higher level of in-phase pattern when participants fixated their focal vision to their partner’s rocking movement compared to peripheral vision (Richardson et al., 2007). It suggests that more visual perceptual information leads to greater extent of coordination. Third, coordination seems inevitable as long as visual perception is available. Issartel et al. (2007) asked participants intentionally not to coordinate while looking at each other’s movement. Results showed that participants’ intrinsic oscillatory frequencies tended to converge when visual information was shared, revealing that they could not avoid influencing each other as soon as visual contact was available (Issartel et al., 2007). In sum, all these studies suggest the importance of visual perception on determining the level of motor coordination. Some studies also focused on the role eye contact plays during social interaction (Wang et al., 2011; Wang and Hamilton, 2014), and found that eye contact facilitated mimicry. Differently, our study tested whether likability influenced IMC simply through looking at the partner’s movement. Moreover, we investigated how natural gaze was oriented in a continuous interpersonal interaction situation. The above-mentioned studies provide reasonable justifications to hypothesize that the amount of gaze targeted onto the partner’s movement determines the level of coordination.

In the experiment reported below, we captured the natural gaze direction of our participants during IMC. Of particular interest was the amount of gaze directed toward the partner’s movement. Eye tracking techniques have been extensively documented as valid tools to detect visual focus (Arolt et al., 1996; Dalton et al., 2005). In our study, in order to ensure that visual perception was the only source of inter-personal entrainment, auditory cues were blocked with proper techniques. Based on the critical role visual perception plays on coupling interactants, we expected that higher level of motor coordination might be attributed to greater amount of visual fixation on the partner’s movement.

## Materials and Methods

### Participants

Twenty-two participants (10 female and 12 male; age 26.9 ± 6.6 years) were recruited from the University of Montpellier and other Universities in Montpellier by asking whether they would like to participate a finger tapping experiment in order to study the individual’s tapping characteristics. Each participant signed the informed consent prior to the start of the experiment. The protocol conformed the Declaration of Helsinki, followed the guidelines of the University of Montpellier, and was approved by the EuroMov IRB. Participants had normal or corrected-to-normal vision, and they were not told about the exact purpose of the experiment until all sessions of the experiment were completed.

### Confederate and Likability Manipulation

A female confederate was employed and conversations were arranged to manipulate the level of likability toward the confederate. An interpersonal finger-tapping task was arranged right after each conversation in order to assess the level of IMC.

The confederate was a 24-year old college female student. She was asked to adopt the similar style of dressing and makeup in order to maintain the identical level of physical attractiveness throughout different likability conditions. In this way, a potential difference in IMC could not be attributed to physical attractiveness, which was reported to influence IMC (Zhao et al., 2015). The confederate was not naïve to the study hypothesis. She was paid for the job and highly motivated to accomplish the task, and she did not know any of the participants.

Three levels of likability were tested: baseline, likeable and unlikeable. The baseline condition captured the first impression, requiring both the confederate and participant to meet and say “Hi” to each other without further communication. In the likable condition, both the participant and the confederate were told to have a conversation on their hobbies and studies. The confederate behaved in a friendly and outgoing manner in order for the participants to like her. She engaged herself completely in the conversation, listening attentively and responding properly to the participant. Her phone was switched off to avoid incoming calls or messages. In the unlikable condition, both persons were asked to have a conversation on controversy topics such as gay marriage. The experimenter indicated in this particular condition that they were allowed to discuss the debated topics. In order to know the participant’s opinion, the confederate always raised the question first. After knowing the participant’s opinion, the confederate intentionally posed opposite opinions. Moreover, she avoided eye contact and acted inattentively when the participant was speaking. To further ensure the success of the unlikable manipulation, the confederate set an alarm on her phone to ring during the conversation (as if a message came in). Afterward she switched off the alarm but continued playing with her phone (as if texting messages). This technique intended to annoy participants to an extent that the likability level would be low. Both people were allowed to ask questions to each other in both likable and unlikable conditions. The conversations in these two conditions lasted around 5 min and the experimenter stopped the conversations at a proper time.

#### Likability Questionnaire

In order to confirm that likability was successfully manipulated, participants rated a likability questionnaire after the conversation in each of the three likability conditions. The questionnaire was tailored by incorporating eight items of the Reysen likability scale. The original Reysen likability scale is an 11-item measurement, and is a valid and reliable tool to assess likability (Reysen, 2005). It uses a 7-point Likert scale format, with -3 representing “strongly disagree” and +3 “strongly agree.” Higher score of all items stands for a higher likability level. The 7th, 10th, and 11th items of the Reysen likability scale were not selected into the likability questionnaire for empirical reasons. For example, the seventh item— “I would like this person as a roommate” — was not chosen because it might have been viewed as inappropriate, especially between a male participant and the (female) confederate. Moreover, the decision of eliminating items was also taken by consulting the questionnaire developer Stephane Reysen, who believed that skipping a few items would not affect the validity and reliability of the questionnaire. In order for the participants to rate their real feeling for the confederate, they were arranged to sit at two corners when filling out the questionnaire so that neither of them knew the other’s appraisal. Meanwhile, they were highly encouraged to rate their genuine feelings. The questionnaire took about 2 min to answer.

### Experimental Procedure

Each participant underwent the three likability conditions. The baseline condition always came before the likable and unlikable conditions, whose order was counterbalanced across number and gender of the participants. Likable and unlikable conditions were conducted at least 2 days apart because the confederate behaved in a completely different way in these two conditions. If both conditions had been arranged on the same day, participants would have been surprised by the great change in the confederate’s attitude, and would thus have been suspicious about the goal of the experiment.

**FIGURE 1:**
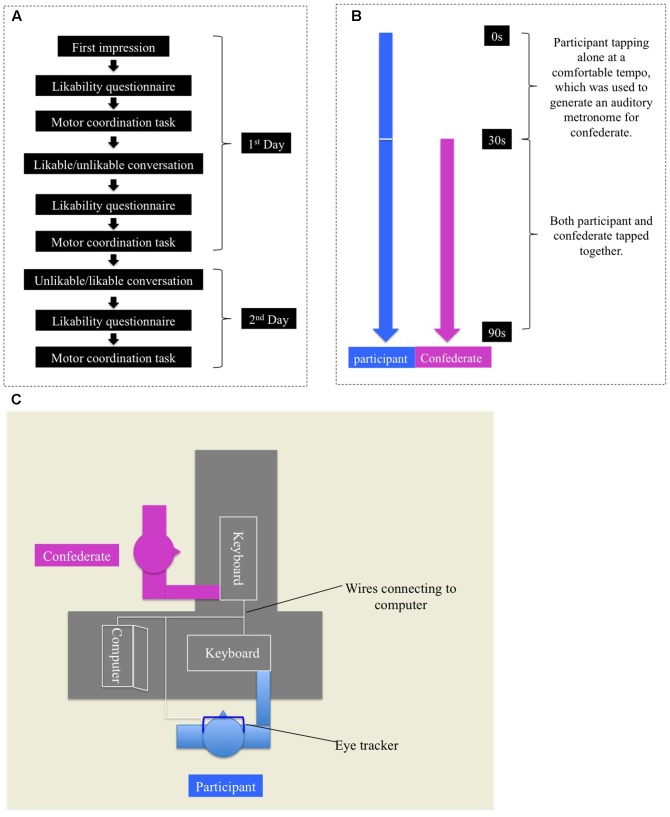
**(A)** Experimental procedure for each participant. Baseline condition came always before the likable and unlikable conditions, whose order was counterbalanced. These two conditions were also separated at least by 2 days. **(B)** Experimental procedure for a single trial of interpersonal finger-tapping task. **(C)** Top-down view of the seats arrangement and experimental materials.

After each likability manipulation and questionnaire, an interpersonal finger-tapping task was conducted to measure the level of IMC in the corresponding likability condition (**Figure 1A**). The tapping task session lasted around 15 min in each condition.

There were six trials of interpersonal finger tapping in each of the three likability conditions. Each trial lasted 90 s, which was composed of two parts – the first 30 s and the last 60 s. The participant tapped alone for the first 30 s, whose data were used to generate an auditory metronome, which beeped at 100% or 150% of the participant’s tapping frequency for the confederate to follow. Then both persons tapped simultaneously for the last 60 s (**Figure 1B**). The 100 and 150% frequencies were repeated three times, and all of these six trials were randomly presented. Participants wore an eye tracker throughout the IMC task. The instruction required participants to tap at a constant and comfortable tempo. They were free to look wherever they wanted, but were instructed not to close their eyes (except for eye blinking) during the IMC task. The confederate was looking straight ahead and was careful to express no emotion during the finger-tapping task. She was particularly instructed to maintain the same performance at all times.

### Apparatus

A Macbook Pro (15-inch, Mid 2012, OSX 10.9.5) connected to two keyboards and an eye tracker (PupilLab©) was used. The Matlab toolbox (Matlab_R2013a) together with Psychtoolbox (Kleiner et al., 2007) were run to generate and deliver auditory metronome to the confederate, to initiate the recording of the eye tracker data, and to collect the tapping data. The confederate and the participant tapped on two separate keyboards, which recorded the finger tapping data. The participant’s keyboard was covered with a shield in order to block the confederate’s peripheral view of their finger tapping. Participants tapped on the “left arrow” key on the participant’s keyboard and the confederate on the “right arrow” on her keyboard.

Gaze direction of the participant was collected with a commercial head-mounted eye tracker, which consisted of two cameras: a scene camera and an eye-tracking camera. The scene camera captured the environmental scene in front of the subject, and the eye camera recorded eye movements. The average recording frequency of both cameras was 30 Hz. The device is a reliable eye-tracking tool for estimating natural gaze direction, with decent temporal-spatial accuracy and precision (Kassner et al., 2014). Data recording was initiated by the first tap of the participant, and was paused manually after each trial was completed. Participants were all naïve with respect to the eye tracking device. They were told that it was used to count the number of eye blinking events during the task. This cover story about the purpose of the eye-tracker was added in order to avoid possible unnatural behavior during tracking behavior.

### Experimental Setup

In the interpersonal finger tapping task, the participant was situated at a 90° angle from the confederate (**Figure 1C**). The particular position ensured that only the participant had a full view of the confederate’s finger tapping, instead of the other way around.

In order to block auditory cues, both the participant and the confederate wore earphones, through which white noise was delivered. Noise was delivered through a cellphone to the participant. As for the confederate, it was delivered together with the auditory stimulus via the computer. The volume of white noise was tuned to an appropriate level, so that it was not uncomfortable but efficient at blocking the tapping sound. Both seats arrangement and white noise were adopted to establish a unidirectional coupling. In such a way, the difference in motor coordination could only be explained by the likability manipulation, and the underlying mechanism could be solely attributed to vision instead of other forms of perception.

In order for the participants not to realize the genuine objective of the experiment, participants were told that they would perform the task together with another participant (in reality the confederate) for the purpose of faster recording experimental data. They were also informed that the computer had assigned the seats randomly, and that it was completely possible for them to remain at the same position throughout the entire experiment. In this case, it was also likely that the same person was wearing the glasses (eye tracker) all the time. As a matter of fact, participants remained in the same seat and wore the eye tracker during all experimental sessions.

At the very end of the experiment, a debriefing was set up by the experimenter to explain to the participants why the confederate acted in such different ways, and to know whether they were aware of the genuine purpose of the experiment. Participants were instructed to not discuss the purpose or the conditions of the experiment during the entire study. Two participants (not included in the 22 Ss) correctly assumed the real objective of the experiment; hence, their data were discarded from further analysis.

### Data Analysis

#### Relative Phase Calculation

In the calculation of relative phase, previous studies examined the distribution of relative phase across the range of 0–180 degrees (Schmidt and O’Brien, 1997; Richardson et al., 2007). It is an efficient way of showing that relative phase values are not evenly distributed, with a dominance around in-phase and anti-phase patterns. However, this methodology helps little to capture how much percentage of in-phase and anti-phase coordination segments occurred in a trial. It incorporates all relative phase values that are lower than 20 degrees in the region of in-phase coordination. But one single point with its relative phase lower than 20 degrees does not necessarily indicate the occurrence of in-phase coordination, and it could also be a sample in the middle of phase drifting. Alternatively, we reckon that in-phase or anti-phase coordination segments are stable periods where relative phase values dwell around these two patterns of coordination. Therefore, in order to detect the intrinsic (i.e., in-phase and anti-phase) patterns of coordination segments, discrete finger tapping data were converted into continuous signals with a sinusoidal function (Varlet and Richardson, 2011).

During the conversion, because both persons performed rhythmic oscillatory movement, two consecutive taps were considered as a full oscillatory cycle, and the position of the tapping moment was set as the value “-1” in the simulated sinusoidal function. Once the continuous signal was obtained, both participant’s and confederate’s signals were filtered by using a second order Butterworth filter, with a cutoff frequency of 10 Hz. Hilbert transform was employed in the final calculation of the relative phase between the participant and the confederate. The first 3 s and the last 2 s were discarded due to the transient process in the beginning and the abnormal value at the end of the Hilbert transform.

#### Dependent Variables of Coordination

We used different variables to compute the coordination level in the 100 and 150% frequency conditions, respectively, due to the fact that both persons tapped at the same frequency in the 100% condition and at different frequencies in the 150% condition. It was indeed not possible to compute in- or anti-phase coordination in the 150% condition.

In the 100% condition, we tested whether the percentage of in-phase, anti-phase, and/or the sum of these two patterns would be higher in the likable condition as compared to the other two conditions. The reason of computing the sum of in- and anti-phase coordination was described in section “Discussion.”

**FIGURE 2:**
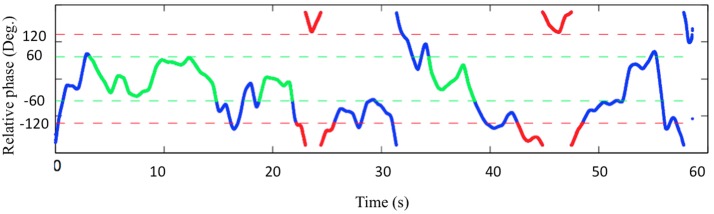
Representative example of the detection of in-phase and anti-phase coordination segments in the 100% frequency condition. Green: in-phase; Red: anti-phase. Blue: neither in-phase nor anti-phase.

The criteria for defining both in-phase and anti-phase patterns of coordination were (1) the existence of a coordination segment no less than five consecutive cycles of tapping, (2) no relative phase value more than 60 degrees deviated from the intrinsic patterns of coordination (**Figure 2**). The criteria were settled empirically to maximally capture the genuine coordination segments and to discard the out-of-coordination segments such as phase drifting. The percentage of coordination was calculated as the ratio of the total length of the specific pattern of coordination relative to the length of the trial.

In the 150% condition, we measured the changes in tapping frequency in the different likability conditions. With the confederate tapping in the participant’s field of view, we expected the tapping frequency of the participant would be entrained to some extent. Specifically, we hypothesized that the participant’s tapping frequency would increase more in the likable condition as compared to the other two conditions. In the unlikable condition, participants might even tap slower because they might intend to be “asynchronous” with the unlikable person (who was tapping much faster in this condition). The tapping frequency change rate was computed as (Freq60 – Freq30)/Freq30, where Freq30 and Freq60 stood for the median tapping frequency during the first 30 s and the last 60 s, respectively.

#### Gaze Direction

**FIGURE 3:**
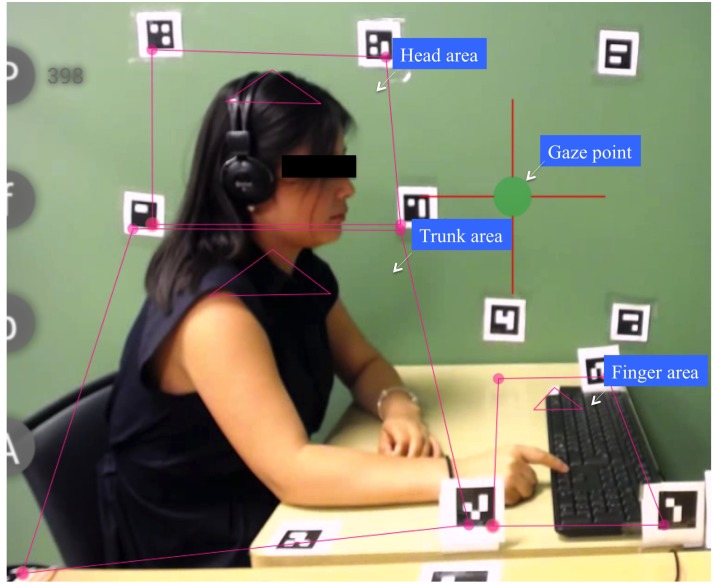
Eye tracker recording interface. Three areas of interest were determined: finger, head, and trunk.

The eye tracker registered the natural visual scan during the motor coordination task. Three areas of interest were defined and examined: head, trunk, and finger (**Figure 3**). The size of these areas was determined with the principle of maximally covering the interested part and excluding extra areas even when the confederate was slightly moving. Of primary interest was whether the amount of gaze direction toward the three defined areas would differ with likableness. For this purpose, we computed the percentage of time when gaze direction was allocated to the interested area during the last 60 s.

Among the three areas, we summed the head and trunk areas together to create a new “body” area since it was difficult to clearly separate these two areas. The confederate’s head was moving occasionally during the coordination task, and it occurred very often that her chin went down into the trunk area.

By examining gaze direction allocated to these two areas – finger and body [termed as Gaze (finger) and Gaze (body), respectively, in the following text] – it was feasible to test whether likability exerted a general effect by looking at the whole body, or it favored the entrainment specifically by looking at the finger area.

#### Expected Results

Due to the facts that most previous studies favored a positive correlation between likability and IMC, and that participants were not instructed to be bond with the confederate, we hypothesized that the coordination level in the likable condition would be higher than in the other two conditions. We also hypothesized that the higher level of coordination would be mediated by a greater amount of gaze direction toward the confederate’s finger. We voluntarily formulated different hypotheses for the 100 and 150% conditions. In the 100% condition, we hypothesized a higher percentage of in-phase, and/or anti-phase, and/or sum of these two intrinsic patterns of coordination in the likable coordination than in the other two conditions. In the 150% condition, we hypothesized a higher frequency increase in the likable condition compared to the other two conditions.

## Results

### Likability Questionnaire

To assess likability through all conditions, the mean of the eight items in the likability questionnaire was calculated. A repeated-measures ANOVA revealed a significant difference for the likability score (*F*_2,42_ = 26, *p* < 0.01, $\eta_{p}^{2}$ = 0.553). The Fisher’s LSD *post hoc* test demonstrated that the level of likability in the likable condition (2.06 ± 0.18) was significantly higher than in the baseline (1.15 ± 0.22) and unlikable conditions (0.688 ± 0.32): both *p* < 0.01; and baseline was significantly higher than the unlikable condition: *p* < 0.05. This result confirmed that the likability manipulation was successfully executed.

### Predicting IMC

In this section, we first built linear mixed-effect models (LMEMs) to explore which predefined factors were significant predictors of the dependent variables by accounting for random effects. We included maximal random effects structure justified by the experimental design and assumptions (Barr et al., 2013). In all of the LMEMs listed below, we specify random slopes for the by-subject effect of Likability and Trial. As a complement to LMEMs, the repeated measures ANOVAs or non-parametric tests were conducted to perform pairwise comparisons. LMEMs were performed by using the package lme4 (Bates et al., 2014) for R (R Development Core Team, 2014), whereas ANOVAs and non-parametric tests were conducted on SPSS (22.0).

#### Testing Gaze as a Mediator between Likability and IMC

As the first step and one of our main objectives, we tested whether Gaze (finger) was a mediator between likability and IMC. According to Baron and Kenny (Baron and Kenny, 1986), at least two prerequisites needed to be fulfilled if gaze (finger) was the mediator between likability and IMC: (1) both Likability and Gaze were independently significant predictors for IMC; (2) only Gaze (finger) but not Likability was significant when both Likability and Gaze were entered into the model to predict IMC. To test prerequisite 1, we built two LMEMs by exploring whether Likability or Gaze (finger) alone exerted significant effect on IMC:

Model A: IMC ∼ Likability
Model B: IMC ∼ Gaze (finger)

In both models, we entered Likability or Gaze (finger) alone as the fixed effect, and participant, participant’s gender, likability order (whether likable was arranged before or after unlikable condition) as random effects. The dependent variables were the occurrence of in-phase, anti-phase and the sum of in-, anti-phase in the 100% condition and the frequency change rate in the 150% condition (**Table 1**). The detection of the significance of Likability or Gaze (finger) was conducted by using the likelihood ratio test (Giampaoli and Singer, 2009). As is shown in **Table 1**, results failed to show that both Likability and Gaze were independently significant predictor for either of the four parameters of IMC. The prerequisite 1 was not fulfilled, indicating that likability alone did not influence IMC, and that Gaze (finger) was not the mediator between Likability and IMC.

**Table 1:** Results of the linear mixed-effect models (LMEMs) predicting interpersonal motor coordination (IMC) (100% condition: occurrence of in-phase, anti-phase and sum of both in- and anti-phase coordination; 150%: frequency change rate) with Likability or Gaze (finger) as the fixed effect.

	Sum (In+Anti)		In-phase		Anti-phase		Frequency change	
				
	*A*	*B*	*A*	*B*	*A*	*B*	*A*	*B*
AIC	1841.6	1845.1	1873.3	1872.4	1572.6	1570.6	1465.2	1460.0
BIC	1900.8	1901.0	1932.5	1928.3	1631.8	1626.5	1524.4	1515.9
Log likelihood	-902.82	-905.53	-918.66	-919.21	-768.29	-768.30	-714.62	-713.0
χ^2^	6.54	1.12	3.40	2.29	1.20	1.17	2.56	5.80
*p*	<0.05^∗^	>0.05	>0.05	>0.05	>0.05	>0.05	>0.05	<0.05^∗^


*Model A: IMC ∼ Likability. Model B: IMC ∼ Gaze. ^∗^*p* < 0.05.*

#### Likability: Trial and Likability: Gaze Interaction Effect on IMC

According to results presented in **Table 1**, our initial hypotheses regarding the effect of likability on IMC and the mediating effect of gaze between likability and IMC seemed to be rejected. This might be because that the effect of likability on IMC varied with time (trial), and/or that the effect of gaze on IMC was moderated by likability. In order to test these possibilities and to explore the effect of other factors on IMC, we created Model C with LMEMs:

Model C: IMC ∼ Likability + Trial + Gaze (finger) + Gaze (body) + Likability:Trial + Likability:Gaze (finger)

In Model C, the dependent variables were the occurrence of in-phase, anti-phase and the sum of in- and anti-phase in the 100% condition, and Frequency change rate in the 150% condition. We entered Likability, Trial, Likability:Trial (interaction), Gaze (finger), Gaze (body), and Likability:Gaze (finger) as fixed effects, and participant, participant’s gender, likability order as random effects. The *p*-value of a fixed effect was determined with the Kenward–Roger approximation to the degrees of freedom (Halekoh and Höjsgaard, 2014).

In the 100% condition, results showed a significant interaction effect of Likability:Trial on the sum of in- and anti-phase coordination (*p* < 0.01), and significant interaction effects of Likability:Trial and Likability:Gaze (finger) on the in-phase coordination (both *p* < 0.05). The statistics approached but did not reached the significant level for the main effect of Likability and the interaction effect of Likability:Gaze (finger) on the sum of in- and anti-phase coordination (0.05 < *p* < 0.1). These results indicated that the effect of Likability on IMC varied with time (Trial), and that likability moderated how Gaze (finger) affected IMC. Further analysis was performed in the following section to seek how IMC varied with Likability and Gaze (finger).

In the 150% condition, results showed that Gaze (finger) exerted a significant effect on frequency change rate (*p* < 0.05).

##### Likability’s effect on IMC in the 100% condition

In order to explore how IMC varied with likability in different trials, we performed the repeated-measures ANOVAs with the structure of 3 Likability (baseline, likable, and unlikable): 3 Trial (first, second, and third trial). The dependent variables were the occurrence of in-phase, anti-phase and the sum of in- and anti-phase coordination.

Results revealed no main or interaction effect of Likability on the occurrence of the in-phase or anti-phase coordination (In-phase: Baseline 34.21% ± 3.51, Likable 32.66% ± 4.43 Unlikable 26.16% ± 3.86. *F*_2,42_ = 1.26, *p* > 0.05, $\eta_{p}^{2}$ = 0.056; Anti-phase: Baseline 10.94% ± 1.77, Likable 8.39% ± 2.20, Unlikable 8.74% ± 1.59. *F*_2,42_ = 0.57, *p* > 0.05, $\eta_{p}^{2}$ = 0.027). However, an interaction effect of Likability:Trial was found for the sum of in- and anti-phase coordination: *F*_4,84_ = 3.45, *p* < 0.05, $\eta_{p}^{2}$ = 0.141. This result was consistent with those obtained with LMEMs shown in **Table 2**. Further, *Post hoc* tests (Fisher LSD) demonstrated that the percentage of the sum was significantly higher in the baseline condition compared to the other two conditions (both *p* < 0.05) in the first trial of the coordination task; and it was significantly higher in the likable condition than in the unlikable condition (*p* < 0.05) in the third trial of the coordination task. The difference between the likable and baseline conditions in the third trial of the coordination task approached but did not reach the statistically significant level (*p* = 0.051).

**Table 2:** Results of the LMEMs for predicting IMC.

	Sum (In+Anti)	In-phase	Anti-phase	Frequency change
Likability	*F*_2,19.60_ = 3.33^∧^	*F*_2,19.72_ = 1.56	*F*_2,19.71_ = 0.47	*F*_2,19.16_ = 1.01
Trial	*F*_2,20.00_ = 0.15	*F*_2,20.06_ = 0.17	*F*_2,20.06_ = 0.74	*F*_2,19.75_ = 0.65
Gaze (finger)	*F*_1,79.48_ = 0.43	*F*_1,79.48_ = 0.43	*F*_1,101.33_ = 1.02	*F*_1,130.81_ = 4.52^∗^
Gaze (body)	*F*_1,71.54_ = 1.40	*F*_1,71.54_ = 1.40	*F*_1,89.37_ = 0.75	*F*_1,85.42_ = 0.09
Likability:Trial	*F*_4,83.24_ = 3.96^∗∗^	*F*_4,83.13_ = 2.74^∗^	*F*_4,83.14_ = 0.96	*F*_4,83.65_ = 0.75
Likability:Gaze (finger)	*F*_2,82.28_ = 3.09^∧^	*F*_2,84.09_ = 3.74^∗^	*F*_2,88.80_ = 0.31	*F*_2,78.80_ = 0.01


*The dependent variables were the occurrence of in-phase, anti-phase and sum of both in- and anti-phase coordination in the 100% condition, and the frequency change rate in the 150% condition. Likability, Trial, Gaze (finger), Gaze (body), Likability:Trial, and Likability:Gaze were entered as fixed effects to see which factor was the significant predictor. ˆ0.05 < *p* < 0.1, ^∗^*p* < 0.05, ^∗∗^*p* < 0.01.*

**FIGURE 4:**
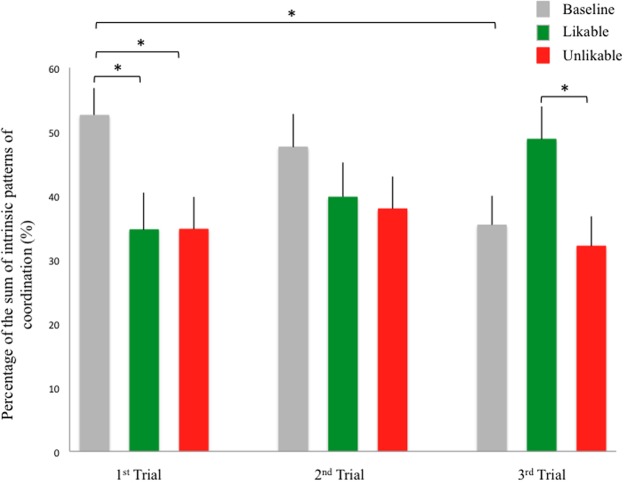
Likability ^∗^ Trial interaction effect on the percentage of the sum of both intrinsic patterns of coordination in the 100% condition. ^∗^*p* < 0.05, ^∗∗^*p* < 0.01. Error bars stand for standard errors.

Examining the coordination change over practice time in each likability condition, we found that the level of coordination dropped significantly in the baseline condition from the first compared to the third trial (*p* < 0.05). It dropped slightly in the unlikable condition and increased in the likable condition, however, the increased level of coordination also approached but did not reach the statistical significance in the likable condition (*p* = 0.084) (**Figure 4**).

In short, our results illustrated that likability led to greater extent of IMC in the last portion of the finger tapping task, and that the level of coordination varied with practice time in the 100% condition.

##### Gaze (finger)’s effect on IMC in 100% condition

The Likability:Gaze (finger) interaction effect on the occurrence of the in-phase coordination (*p* < 0.05) as well as the sum of the in- and anti-phase coordination (0.05 < *p* < 0.1) indicated that likability moderated the impact of gaze (finger) on IMC, suggesting that looking at the confederate’s finger exerted different effect on IMC depending on the level of likability. In order to understand the relation between Gaze (finger) and IMC in each of the likability conditions, we checked the correlation between Gaze (finger) and IMC (in-phase and sum of in- and anti-phase) in these three conditions independently.

As for the relation between Gaze (finger) and the occurrence of in-phase coordination, results showed a positive correlation between these two variables in the likable condition (*r* = 0.377, *p* < 0.05), but not in the other two conditions (baseline: *r* = -0.051, *p* = 0.671, unlikable: *r* = -0.135, *p* = 0.271). The comparison between the three correlational strengths was performed (Raghunathan et al., 1996) to show that the correlation was significantly stronger in the likable condition as compared to the baseline (*z* = 2.50, *p* = 0.012) and to the unlikable conditions (*z* = 3.12, *p* = 0.002). No significant difference was revealed between baseline and unlikable condition (*z* = 0.47, *p* = 0.64).

**FIGURE 5:**
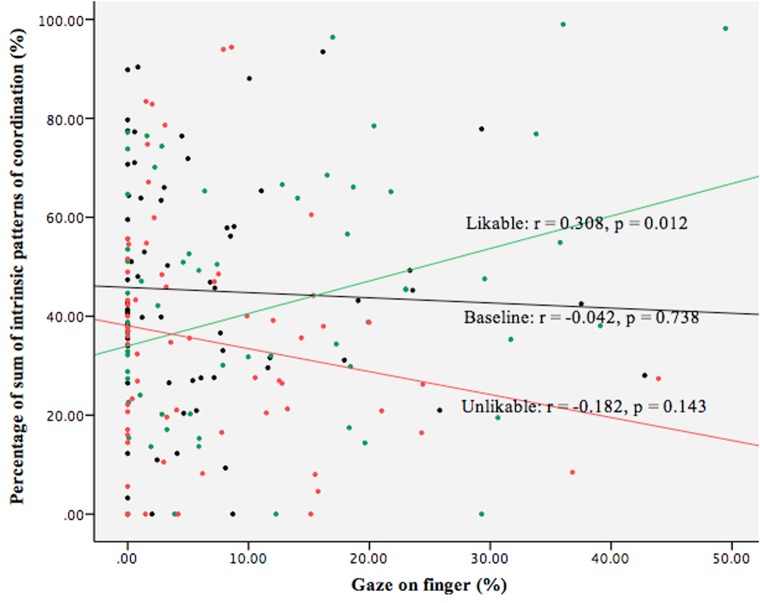
Scatter plot and linear correlation between interpersonal motor coordination (IMC) and gaze on finger in the 100% condition. For brevity, only the relation between the amount of gaze on finger and the occurrence of sum of in- and anti-phase coordination was plotted. Black dots: baseline; Green: likable; Red: unlikable.

Similar results were obtained for the correlation between Gaze (finger) and the sum of the in- and anti-phase coordination (baseline: *r* = -0.042, *p* = 0.738; likable: *r* = 0.308, *p* < 0.05; unlikable: *r* = -0.182, *p* = 0.143; **Figure 5**). The comparison tests also showed that the correlation in the likable condition was significantly higher than the baseline (*z* = 1.99, *p* = 0.046) and unlikable condition (*z* = 2.25, *p* = 0.024), and no significant difference between baseline and unlikable condition (*z* = 0.14, *p* = 0.89). All these results suggested that focal visual information uptake of the partner’s movement led to IMC only when the interaction partner was likable.

##### Gaze (finger)’s effect on frequency change rate in 150% condition

In the 150% condition, the LMEM showed that likability was not a significant predictor of the frequency change rate (Baseline: 4.3% ± 1.23, Likable: 4.3% ± 1.54, Unlikable: 2.3% ± 1.39), and that only Gaze (finger) was a significant predictor. Pearson’s correlation was run to show that Gaze (finger) was significantly positively correlated to the frequency change rate in the 150% condition (*r* = 0.158, *p* < 0.05), suggesting that looking at the confederate’s finger tapping would increase the participant’s tapping frequency regardless of the level of likability.

### Likability’s Effect on Gaze (Finger)

In this step, we also explore whether likability would influence the amount of gaze onto the confederate’s movement. We used LMEM to predict the amount of Gaze (finger) by entering Likability, Trial, and Likability:Trial as fixed effects, using participant, participant’s gender, and the likability order as random effects. Results showed that in both 100 and 150% conditions, Likability was not a significant predictor (100%: *F*_2,20_ = 3.08, *p* > 0.05; 150%: *F*_2,20_ = 2.01, *p* > 0.05). The Friedman’s tests also failed to show a significant difference in the amount of Gaze (finger) between different likability conditions (100%: Mdn_Baseline = 2.9%, Mdn_Likable = 5.5%, Mdn_Unlikable = 3.0%, *p* = 0.277; 150%: Mdn_Baseline = 1.7%, Mdn_Likable = 5.0%, Mdn_Unlikable = 1.4%; *p* = 0.203). These results indicated that the amount of gaze onto the confederate’s finger did not depend on the likability level.

### Results Summary

(1) Post-conversation likability questionnaire showed that the level of likability was highest in the likable condition and lowest in the unlikable condition with baseline in the middle. It suggested that the manipulation of likability was successfully performed.
(2) In the 100% condition, our results demonstrated that likability’s effect on IMC varied with time. Baseline condition yielded the highest level of IMC at the first portion of time, and likable condition the last portion. Furthermore, our results showed that gaze onto the confederate’s finger was not a mediator between likability and IMC. Instead, we found that looking at the confederate’s movement resulted in coordination with her only in the likable condition.
(3) In the 150% condition, results failed to show the effect of likability on the frequency change rate. The frequency increase rate was found to be positively correlated to the amount of gaze onto the confederate’s finger tapping.
(4) Our results indicated that looking at the confederate’s body did not influence IMC performance, and its effect on IMC was not discussed further. Moreover, participants cast literally equal amount of gaze onto the confederate’s finger in all likability conditions.

## Discussion

Our study explored whether likability influences how individuals behaviorally coordinate with each other while exploring natural gaze direction. We found that when the confederate was tapping at the same tempo as the participant, likability affects IMC in interesting ways over time: While likability had no influence on IMC early in the motor synchronization task, we saw that participants who liked their partners — due to an induced friendly conversation in the interaction — showed higher IMC as the interaction wore on, compared with participants who had neutral or unfriendly interactions with their partner. More interestingly, we found that likability of the partner moderated how focal visual information uptake influenced IMC.

Previous studies demonstrated that vision is an essential element in coupling two individuals (Schmidt and O’Brien, 1997; Richardson et al., 2007; Oullier et al., 2008), and the amount of available visual information is positively correlated to the level of unintentional coordination (Richardson et al., 2007). The conclusion is drawn without taking into account the likability of the coordination partner, which is a key psychosocial feature of the person. Differently from these findings and as a novelty of our study, we found a positive correlation between focal visual information uptake and IMC only when her likability was high, inferring that whether looking at the partner’s movement would lead to coordination depends on the likability of the person. It implied that likability of the interaction partner might have been a confounder in those studies, suggesting that likability needs to be seriously treated in future studies on IMC. Furthermore, the moderating effect of likability determined the participant’s tendency to coordinate more when the partner was likable, and it helped partially to explain the higher level of coordination in the last portion of the task in the likable condition.

We noticed, however, that the variations of gaze (finger) as a function of likability with time did not correspond exactly to that of IMC in the 100% condition, indicating that gaze alone could not explain the performance of IMC. It was still impossible, however, to deny that IMC was determined by the amount of visual information uptake since vision incorporates both focal and peripheral vision. In the present study, our eye tracker only registered focal vision, and did not take into account the peripheral view. Recent studies instructing the participant’s vision not to be focused on the coordination object suggest that peripheral vision would also lead to some level of coordination (Richardson et al., 2007), particularly when the object was oscillating at the same frequency. Similarly, our results also indicated that participants coordinated with the confederate by using the peripheral information. As shown on **Figure 3**, the “finger” area was restricted to a confined area, and it was not located in the natural straight visual field of the participants. Moreover, there were several cases in which even if gaze direction was not directly focused on the confederate’s finger, the level of IMC was high (**Figure 5**). This observation was a demonstration that the participant’s peripheral vision captured the confederate’s tapping information; otherwise no IMC could be established in the current study’s paradigm, since no other forms of perceptual information (aside from visual information; e.g., sound, touch) about the partner were available to the participant (Richardson et al., 2007). Therefore it could only be declared that the focal visual information uptake was not the mediator between likability and IMC. Further investigation is needed concerning how the focal and peripheral visual information uptake influence IMC.

As for the underlying reason for why IMC varied with likability and time, apart from the moderating effect of likability, we assumed that motivation might have also been involved in the interplay between likability and IMC. This was mainly because of our findings that IMC was high in the first trial of the baseline condition, and in the third trial of the likable condition. The first trial of the baseline condition was always arranged right after the participant and the confederate first met. Participants might have been curious about the confederate; hence, they were probably motivated to have a further interaction with her. As motor coordination serves as a useful tool to establish affiliation (Lakin and Chartrand, 2003; Hove and Risen, 2009), the high level of coordination at the beginning may manifest the participant’s motivation to be affiliated with the confederate (Miles et al., 2011). The decreasing trend of IMC in the baseline and unlikable conditions might be due to a general decrease in motivation with time, although participants were only engaged in tapping for a total of 4.5 min during each of the three visits to the lab. In the likable condition, however, the decrease was compensated by the confederate’s likableness. The finding was consistent with our expectation. It corresponds to our daily experience that interaction with unlikable people is shortened, leading to a reduced amount of coordination. On the contrary, with persons we genuinely like, we attempt to maintain affiliation, and this may lead to a persistent high level of motor coordination since motor coordination is able to increase affiliation (Hove and Risen, 2009). However, the claim that motivation genuinely played a role in this process obviously needed further investigation. For instance it will be helpful in future research to record the level of motivation trial by trial in order to seek whether changes in IMC can be explained by motivation.

When the confederate tapped at 1.5 times of the participant’s frequency, our results demonstrated a positive correlation between the amount of gaze on the confederate’s movement and the frequency change rate regardless of the likability level. This finding was consistent with Issartel et al.’s finding that available visual information could lead to frequency entrainment no matter if they were willing to coordinate or not (Issartel et al., 2007). But our initial hypothesis was rejected that participants did not show higher amount of frequency increase when the level of likability was high. One possible reason might be that during the IMC task, participants did not look more onto the confederate’s tapping in the likable condition as compared to the other two conditions. Because the extent of frequency increase only depended upon the amount of focal vision on the movement in this particular condition, this determined that the frequency increase rate was not different in these likability conditions.

To be noticed is that our results showed that likability moderated the relation between gaze (finger) and IMC in the 100% but not 150% condition. It was not clear why the moderation effect of likability occurred only when the partner was oscillating at the same tempo. It might be due to the nature of the coordination task since past research showed that the level of coordination differed with task and that people were less entrained when the partner’s oscillating frequency exceeded their own preferred frequency (Richardson et al., 2007). Previous findings adopting the unintentional IMC paradigm suggested that individuals are more likely to be unintentionally entrained into coordination regimes when the tapping frequency is within the range of ±10% of one’s preferred frequency (Schmidt and Richardson, 2008). In our experiment, the confederate was tapping at 1.5 times of the participant’s current tapping frequency, which could be perceived as too high as compared to their own tempo. In order to follow the instruction of “keeping a constant” frequency, they might have restricted themselves to be influenced by the confederate. Therefore we reckoned that the effect of likability might have been masked by the participant’s willing to follow the instruction of maintaining their own tempo.

In the 100% condition, we checked the occurrence of in-phase, anti-phase and the sum of these two patterns in our study, whereas previous studies treated the occurrence of in- and anti-phase separately (Schmidt and O’Brien, 1997; Richardson et al., 2007). Some only calculated in-phase coordination. For example, Hove and Risen (2009) found the effect of phase entrainment on the likability ratings. Phase relation was only referred to in-phase coordination since the synchrony was calculated as the co-occurrence of the two person’s taps within 100 ms in their study (Hove and Risen, 2009). In our study, we computed the sum of in- and anti-phase patterns. The phenomenon that individuals are entrained into these two patterns of coordination could be explained by two main theories. In one theory, the finding of mirror neurons might be effective in explaining why people are engaged into in-phase coordination (performing the same movement) (Rizzolatti et al., 1996). However, it does not well explain anti-phase coordination since anti-phase coordination requires individuals to perform a temporally opposite movement. We believe that the second theory, the ecological approach to perception and action, provides a more reasonable account. According to this approach, a person is able to directly perceive both the environment and the self in relation to the environment (Gibson, 1979). Existing work evidenced that relative phase (an index of the relation between self and the environment) exists in the visual information that could be directly harnessed to coordinate with the perceived movement (Schmidt et al., 1990; Bingham et al., 1999). The main reason why individuals are entrained into in- and anti-phase coordination might be because the near-preferred-frequency rhythmic movement contains particular visual information that triggers individuals to spontaneously perform corresponding coordinating behavior. The characteristic of being triggered by external stimuli (be it social or not) might represent one’s overall sensitivity to the visual information of the external stimuli. Studies on mimicry suggest that the general sensitivity is critical for establishing affiliation with others (Chartrand and Bargh, 1999; Lakin and Chartrand, 2003), and it may also be affected by one’s personality traits (e.g., pro-social trait, extraversion) or clinical diagnosis (e.g., autism, depression) (Condon and Ogston, 1966; Lumsden et al., 2012; Marsh et al., 2013; Duffy and Chartrand, 2015). Therefore, if we consider both in- and anti-phase patterns of coordination as representing one’s general sensitivity to the visual information, it is not unreasonable to take the sum of these two intrinsic patterns together as an index of the level of coordination. Moreover, taking the sum of both in- and anti-phase together did not violate the results of previous work (Schmidt and O’Brien, 1997; Richardson et al., 2007), in which the sum of these two patterns of coordination was also statistically higher than the chance level.

In sum, our study indicated that the coordination task itself influenced how individuals behave. The effect of likability only becomes obvious when the coordination partner was oscillating at one’s preferred frequency. Our study explored the natural gaze direction during the coordination task, and it inferred the importance of investigating the role of peripheral vision and motivation during the interaction. Overall, our study suggests that IMC is a complex phenomenon, which is sensitive to multiple factors.

### Strengths and Weaknesses

Our study adopted a within-subject design by having participants interact with the same person in three different likability conditions, which simulated the real social situation in a good way, because it occurs in our daily life that likability of the same person can change with time and events. It is argued that if IMC varies with likability even with the same person, it is possible to assess the level of likability through measuring the performance of IMC. This particular experimental design might provide empirical evidence particularly for people who are interested in evaluating interpersonal relationship through behavioral assessment.

Motivated by previous studies reporting the close relation between visual perception of the partner’s movement and IMC, we explored how the participants directed their gaze during the coordination task. Different from studies which required participants to close their eyes or look in a specific direction (Richardson et al., 2007; Oullier et al., 2008). Our present study released the visual constraints by allowing participants a natural looking behavior. Together with other studies investigating natural gaze during interaction (Broz et al., 2012; Gironzetti et al., 2016), our study served as an expansion for seeking natural gaze in IMC specifically.

One weakness lies in the lack of naturalness of the IMC task. Here we adopted a finger-tapping task, which is not a common daily human activity. Recent studies based on the advancement of image analyzing techniques evidenced the possibility of measuring coordination in more natural settings (Ramseyer and Tschacher, 2011; Schmidt et al., 2012, 2014; Paxton and Dale, 2013; Kupper et al., 2016). Kupper et al. (2016) used the motion energy analysis to obtain the time series of the activity of a pre-defined area of a person during a conversation by means of detecting pixel changes between two consecutive images (Ramseyer and Tschacher, 2011; Kupper et al., 2016). Their studies indicated that this technique is a valid tool to capture the coordination level during natural social interactions. Schmidt et al. (2012, 2014) implemented a similar image analyzing technique to compare the phase relation between the two time series in a joke-telling task, and found the dominant presence of intrinsic in-phase and anti-phase patterns of coordination. Paxton and Dale (2013) recorded how participants interacted during conversations and analyzed their bodily synchrony with frame differencing analysis. Complexity matching was also reported as a means to capture coordination in a natural dyadic conversation (Abney et al., 2014). In addition, Grammer et al. (1998) reported using behavioral pattern searching algorithms to look for behavioral correlates of coordination during natural conversation. These studies showed the possibility of directly measuring coordination in an ecological setting. However, implementing purely natural conversational situations in our case would pose a considerable difficulty to reveal whether the level of coordination was influenced by the amount of perceptual information uptake. First, interactants are moving in a gross way during natural interaction, and exhibit simultaneously various gestures, postural sway, head movements and so on. Second, eye tracking does not guarantee a specific relation between gaze direction and source of entrainment, as stated above. Third, the control of other types of perceptual information uptake, such as auditory perception, is difficult to achieve during natural conversation. In our study, we tried to adopt the best compromise between task naturalness and mechanism exploration.

Due to technical problems, although the recording of the eye tracker was intended to be launched by the first tap of the participant, sometimes the eye tracker was initiated a bit late (within 2 s). In this case, we checked the overall distribution of the coordinated behavior instead of the moment-to-moment dynamics of coordination. This limitation prevented us from exploring the hypothesis whether gaze onto the partner’s movement preceded the coordinated behavior.

Another issue pertains to the ongoing concern regarding the use a confederate in our experiment. Recent studies indicated that the confederate’s behavior can be different from the spontaneous behavior of naïve participants because they are familiar with the study hypothesis and procedure (Brennan et al., 2010), and this might influence the results. A recent meta-analysis also found that involving confederates in the experiment might influence how participants perceive them and the relationship between them (Vicaria and Dickens, 2016), which might affect the IMC performance. The confederate was aware of the hypothesis in our study, and this might have affected the participant’s performance in IMC although we tried to reduce the possibility to the minimal level. In the experiment, she was specifically instructed to express a neutral emotion when tapping with the auditory metronome in all conditions. Considering the simplicity of the task the confederate was performing (finger tapping without communicating with the participant), we assumed that her performance in the interpersonal finger tapping task could be literally considered as equivalent in different likability conditions. In this sense, the difference in IMC between likability conditions might not be attributed to the employment of the confederate. Even though, setting another condition with a naïve participant might be ideal to determine whether the employment of the confederate affected our results.

## Conclusion

As human behavior could be both the output of the cognitive processes and the vehicle one uses to achieve one’s purpose, the impact of likability on IMC is more than straightforward. Individuals may coordinate at both high and low level with a likable person depending on multiple factors such as likability, motivation, gaze direction, and so on. Our study indicates that psychosocial properties such as likability of the interaction partner should be cautiously treated when investigating IMC.

## Author Contributions

ZZ, RS, LM, MG, and BB designed the experiment. ZZ and RS wrote the matlab code for running the experiment. ZZ, RS, LM, and BB analyzed the data. ZZ, RS, LM, and BB wrote the manuscript.

## Conflict of Interest Statement

The authors declare that the research was conducted in the absence of any commercial or financial relationships that could be construed as a potential conflict of interest.
